# Cost-utility analysis of telitacicept versus belimumab in the treatment of systemic lupus erythematosus in China

**DOI:** 10.3389/fpubh.2025.1712454

**Published:** 2026-01-15

**Authors:** Chang Liu, Libo Tao, Yi Yan, Yao Wu, Fangxu Wang, Shuang Sun

**Affiliations:** Center for Health Policy and Technology Evaluation, Peking University Health Science Center, Beijing, China

**Keywords:** belimumab, cost-utility analysis, pharmacoeconomic, systemic lupus erythematosus, telitacicept

## Abstract

**Objective:**

Telitacicept and belimumab are the only two approved biologics in China for treating systemic lupus erythematosus (SLE). With the widespread clinical use of biologics, more reliable clinical evidence has been provided. This study seeks to evaluate the cost-effectiveness of two biologic treatments for SLE in China by utilizing current medication prices and the most recent clinical research.

**Methods:**

A cost-effectiveness analysis comparing telitacicept and belimumab was conducted by developing a lifetime SLE partition survival model using the Systemic Lupus Erythematosus Disease Activity Index 2000 (SLEDAI-2 K) score, which considers the relationship between organ damage and death. The data were extracted from the literature with model assumptions. Scenario analysis of short term treatment (5 years) and typical one-way and probabilistic sensitivity analyses were performed.

**Results:**

After lifetime simulation, compared to belimumab treatment, telitacicept treatment can save a total of ¥57751.00, including expenses of drug usage and treatment of complications. Meanwhile, it gained 0.499 quality adjusted life years, resulted in a negative ICER. Telitacicept is more cost-saving than belimumab (Dominant).

**Conclusion:**

Under current pricing, treatment settings and efficacy data in China, telitacicept demonstrates superior cost-effectiveness compared with belimumab for long-term SLE management by reducing medication costs while delivering additional health benefits.

## Introduction

Systemic lupus erythematosus (SLE) is a systemic autoimmune disease characterized by multi-organ involvement, alternating periods of relapse and remission, and the presence of abundant autoantibodies (1–3). The current estimated number of SLE patients nationwide ranges between 700,000 and 1 million (4, 5). In China, the incidence rate of SLE is approximately 14.09 per 100,000 population, with a prevalence of approximately 47.53 per 100,000 (6, 7). SLE patients face significant health deterioration and disease burden. Without timely treatment, SLE will cause irreversible damage to the affected organs, reduce quality of life, which further elevates their risks of rehospitalization and mortality (8–10). Regarding healthcare costs, SLE management typically requires long-term medication to control disease activity.

Biologics are recommended for the treatment of SLE when patients fail to achieve treatment targets or experience disease relapse after hormone and conventional immunosuppressant therapy (7). Telitacicept and belimumab are the only two approved biologics in China for treating SLE. Both have been included in the National Basic Medical Insurance. Telitacicept is a novel biologic agent independently developed in China. It was approved in 2021 for the treatment of systemic lupus erythematosus (SLE). Available clinical evidence demonstrates that both telitacicept and belimumab are effective in reducing SLE disease activity, with comparable safety profiles. Compared with belimumab, telitacicept appears to demonstrate slightly superior efficacy, as reflected by higher rates of Lupus Low Disease Activity State (LLDAS) achievement and Systemic Lupus Erythematosus Responder Index-4 (SRI-4) response, as well as a greater reduction in the Systemic Lupus Erythematosus Disease Activity Index 2000 (SLEDAI-2K) score (11–13). Furthermore, in SLE patients with renal involvement, telitacicept showed a more pronounced effect in reducing immunoglobulin G (IgG) levels and improving the rate of complete renal response (14, 15). In China, the unit price of telitacicept is higher than that of belimumab. The prescribing information for both drugs recommends an initial full-dose regimen followed by dose reduction upon achieving stable disease. In both treatment phases, the total course cost of telitacicept is lower than that of belimumab.

With increasing clinical use of telitacicept, more evidences have been published for these two drugs. This study aims to compare the cost-utility of these two drugs for treating SLE based on current drug prices and the latest clinical evidence.

## Materials and methods

### Study design

Based on recent clinical findings and drug prices, this study explored the differences in cost and health outcomes between telitacicept and belimumab for treating SLE. A partition survival model was developed based on the key therapeutic effect index to systematically evaluate the long-term cost-utility from a health system perspective. All parameters for the model were extracted from published clinical studies (11–13) that compared telitacicept and belimumab. Based on these studies, patients entering this model were assumed to be 31 years old, with an SLEDAI-2K score of 9.94. Patients in the telitacicept group were assumed to receive a telitacicept injection of 160 mg weekly for the first 6 months, followed by a 50% dose reduction according to the drug label and clinical expert consultations. Patients in the belimumab group were assumed to receive belimumab intravenous injection 600 mg per dose, administered every 2 weeks for the first 3 doses, followed by every 4 weeks after that.

### Model structure

Through a literature review and expert consultation, an SLE disease model was constructed based on SLEDAI-2K score, a widely recognized efficacy measure that comprehensively reflects SLE disease activity (16). The SLEDAI-2K is used to assess disease activity in patients with SLE over the preceding 10 days. It quantifies disease activity by scoring 24 weighted, disease-related clinical and laboratory manifestations across nine organ systems and laboratory domains. Each manifestation is assigned a predetermined weight (ranging from 1 to 8 points) and is assessed in a binary (present/absent) manner. The total score ranges from 0 to 105. A lower score reflects lower disease activity, greater clinical stability, or a state of remission.

**Figure 1:**
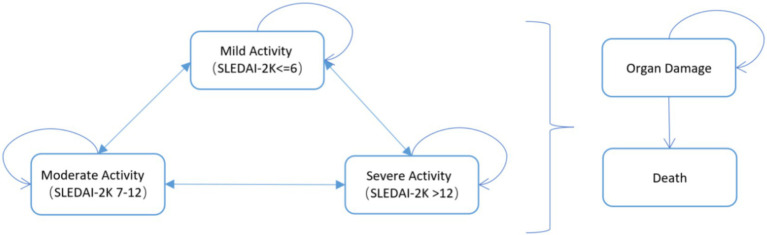
Partitioned survival model for systemic lupus erythematosus.

The partitioned survival model for SLE is presented in Figure 1, with disease activity categorized into three states based on SLEDAI-2K scores: mild (≤6 points), moderate (7–12 points), and severe (>12 points), allowing for transitions between these states. Each model cycle accounted for patients developing organ damage or death, with the risk varying by disease severity, while the organ damage state represented a weighted composite of multiple organ involvements. Considering the progression of SLE and clinical monitoring intervals, the model was structured with a 1-month cycle length over a lifetime horizon [48 years, as the initial age in model is 31 and the current average life expectancy in China is 79 years (17)].

### Health outcomes

**Figure 2:**
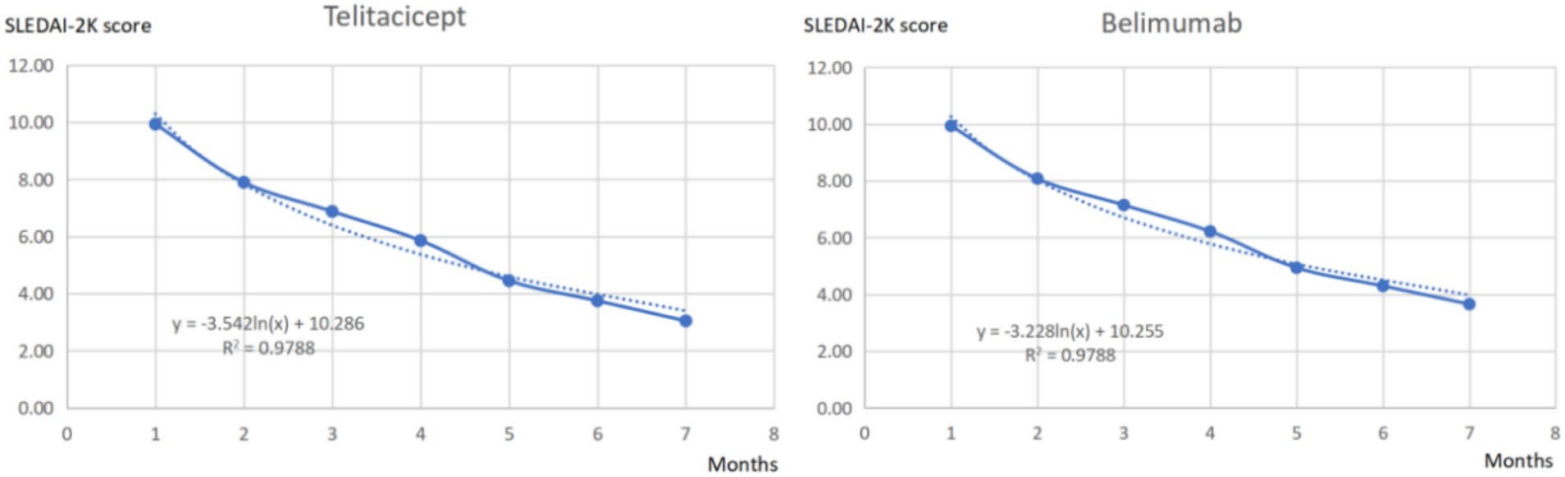
Fitted results of post-treatment SLEDAI-2K score changes.

A comprehensive search identified three high-quality clinical studies (11–13) comparing telitacicept and belimumab, all conducted in Chinese populations with follow-up periods of ≥24 weeks. The post-treatment SLEDAI-2K score changes at 24 weeks and their trends from these studies were integrated for curve fitting, generating the score distribution per cycle for both treatments. Curve fitting was performed through an initial visual assessment of the scatter plots, followed by a quantitative evaluation incorporating the statistical metrics Akaike Information Criterion (AIC) and Bayesian Information Criterion (BIC). Five candidate distributions—namely, the exponential, Weibull, Gompertz, log-logistic, and lognormal distributions—were tested. The lognormal distribution demonstrated the smallest AIC and BIC values, indicating that it provided the optimal fit. Thus, the lognormal distribution was selected for the final curve fitting in this study. Analysis of the 24-week treatment outcomes showed the telitacicept group achieved greater SLEDAI-2 K score reduction (6.89 points) compared to belimumab (6.28 points), with curve fitting results detailed in Figure 2.

Using data from the Johns Hopkins Cohort, the monthly organ damage and death probabilities of mild, moderate, and severe states were derived based on the disease severity correlated risks (18) (Supplementary Table 1). The health utility values of mild, moderate, and severe states were extracted from Parodis’s study (19) (Supplementary Table 2). The parameters used are provided in Table 1. The utility reduction of organ damage was sourced from Bindra’s study (20). Specific organ damage involvement and utility reductions are presented in Table 2.

**Table 1:** Probability of severe events and utilities of each health state.

Health states	Probabilities of organ damage	Probabilities of death	Utilities
Mild activity (SLEDAI-2K < =6)	0.00619	0.00100	0.690
Moderate activity (SLEDAI-2K 7–12)	0.00763	0.00104	0.631
Severe activity (SLEDAI-2K > 12)	0.01235	0.00111	0.568

**Table 2:** Organ damage annual costs, disutilities and percentage.

Organ damage	Annual costs	Disutilities	Percentage
Cardiovascular	38,321	−0.076 (28)	7.14%
Gastrointestinal	47,167	−0.24 (29)	2.14%
Musculoskeletal	28,839	−0.03 (30)	52.98%
Neuropsychiatric	40,069	−0.64 (31)	6.47%
Ocular	35,918	−0.029 (32)	1.24%
Peripheral vascular	34,432	−0.076 (28)	51.69%
Pulmonary	53,274	−0.327 (33)	0.96%
Renal	35,879	−0.26 (34)	38.53%
Skin	29,231	−0.11 (29)	64.90%
Total	73166.67	−0.2822156	

### Costs

The lifetime treatment costs for both drugs were calculated based on the drug label and clinical use. The details are shown in Table 3. Although studies showed no statistically significant difference in drug-related adverse events (11), the adverse event management costs were still included in the calculations, as shown in Table 4. The costs of organ damage management were sourced from a Chinese SLE patient burden study (21) based on the Chinese SLE Treatment and Research Group (CSTAR) database. Specific organ damage involvement and management costs are presented in Table 2.

**Table 3:** Costs of telitacicept and belimumab.

Drugs	Price (¥/vial)	Monthly cost 1	Monthly cost 2	Monthly cost 3	Package insert	Settings
Telitacicept	777.86 (80 mg)	6222.88	3111.44	—	The recommended dose is 160 mg per administration, to be given once weekly via subcutaneous injection. Preferred injection sites include the thigh, abdomen, and upper arm. During treatment, the dose may be reduced to 80 mg per administration based on the clinician’s assessment of the patient’s safety and tolerability.	Monthly cost 1 for the first 6 months, followed by monthly cost 2 after that
Belimumab	700 (120 mg)	7000.00	5250.00	3500.00	The recommended dosing protocol is 10 mg/kg, administered every 2 weeks for the first 3 doses, followed by every 4 weeks after that.	Monthly cost 1 for the first month, Monthly cost 2 for the second month, followed by Monthly cost 3 after that

**Table 4:** Incidence and management costs of adverse events.

Adverse events	Belimumab	Telitacicept	*p*-value	Management costs
Infection	24 (24.0%)	25 (24.8%)	0.90	100
Gastrointestinal disorders	1 (1.0%)	2 (2.0%)	1.00	40
Diarrhea	4 (4.0%)	1 (1.0%)	0.21	20
Leukopenia	4 (4.0%)	5 (4.8%)	1.00	0
Total costs	25.20	25.80		

### Cost-utility analysis

A cost-utility analysis was carried out to compare telitacicept to belimumab over a 48-year (lifetime) time horizon calculation. In accordance with Chinese guidelines, a discount rate of 5% was applied to both costs and utilities, with 0–8% considered in the sensitivity analysis. The incremental cost-effectiveness ratio (ICER) was calculated by dividing the difference in mean costs between the two strategies by the difference in mean quality adjusted life years (QALYs) gained. This ICER represents the extra cost that must be spent to gain an additional QALY with the telitacicept treatment. 1–3 times China’s per capita GDP was used as the willingness-to-pay (WTP) threshold to assess the cost-effectiveness of telitacicept.

### Scenario analysis

Since the lifetime horizon setting may cause bias, we conducted a short-term simulation with a 5.4 year time horizon. The selection of a 5.4 year period was based on the longest follow-up data currently available from a belimumab clinical trial (22), which reported a mean treatment duration of 5.4 years among 1,304 enrolled SLE patients. Additionally, by back-calculating the model results, we estimated the minimum treatment duration at which the total healthcare costs between the two treatment groups would be equal and at which the ICER would meet the one times China’s per capita GDP threshold.

### Sensitivity analysis

To assess the uncertainty of the results, one-way sensitivity analysis was performed. Based on the health economics evaluation sensitivity analysis methods, a one-way sensitivity analysis was conducted by adjusting cost parameters and effect parameters (organ damage and death probabilities, health utility values) by±20% based on common practices of pharmacoeconomic sensitivity analysis. For parameters ranging from 0 to 1 (utility values, discount rates, adverse event rates, organ damage incidents), values exceeding 1 were set to 1 for calculation. The results from those analyses are visually in a tornado diagram, sequentially highlighting variables with the most significant impact on the cost-utility outcomes.

A probabilistic sensitivity analysis (PSA) was carried out using a Monte Carlo simulation and calculated new ICERs in 5000 random resamples. The distributions of each variable were set based on previous research experience. Gamma distributions were assumed for the cost data, beta distributions for probabilities and utilities and normal distributions were assumed for the mean and standard deviation that defined the log-normal curve (Supplementary Table 3). To measure the uncertainty around ICER, 95% CIs around the estimates of incremental cost and incremental effectiveness were calculated. Scatter plots were constructed and used to construct a cost-effective acceptability curve, showing the probability that telitacicept would be cost-effective for different levels of willingness to pay by the decision-maker.

## Results

### Cost-utility analysis at the lifetime

This study assumed that 1,000 patients with an initial SLEDAI-2 K score of 9.94 enter the telitacicept and belimumab groups with equal probability. The evaluation model ran for 48 years (lifetime). Over a lifetime horizon, telitacicept demonstrated a greater reduction in the SLEDAI-2K scores (mean difference: 1.96 points) compared to belimumab. As the model simulation results demonstrated, compared to belimumab lifetime treatment, telitacicept lifetime treatment can save a total of ¥57751.00, including expenses of drug use and organ damage management, meanwhile gained an additional 0.499 QALYs, resulted in a negative ICER. It demonstrated that telitacicept is more cost-saving than belimumab (Dominant).

### Scenario analysis

Short-term modeling (the evaluation model ran for 5.4 years) results demonstrated that compared to belimumab, telitacicept treatment can save a total of ¥8458.04, meanwhile gained an additional 0.135 QALYs. Short-term modeling also resulted in a negative ICER, demonstrating that telitacicept is cost-saving (dominant) compared to belimumab.

At a treatment duration of approximately 3.08 years, total healthcare costs become equivalent between two groups. Beyond this point, telitacicept is dominant (lower cost, superior efficacy). At a treatment duration of approximately 1.92 years, the ICER reaches one times China’s per capita GDP. Beyond this point, and at this GDP-based threshold, telitacicept is identified as the cost-saving option.

### Sensitivity analysis

**Figure 3:**
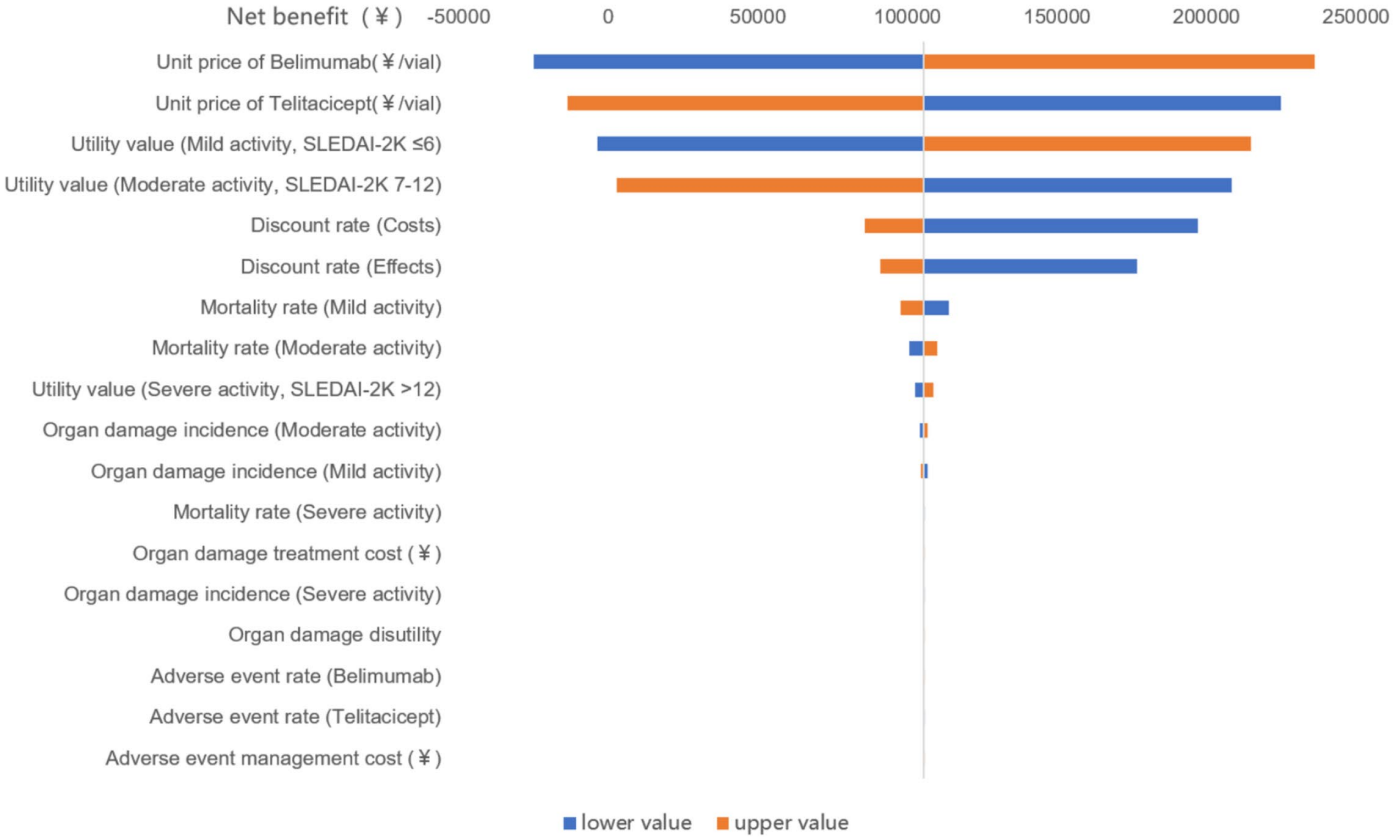
One-way sensitivity analysis results.

The one-way sensitivity analysis results showed that the cost parameters of both treatment groups were the most sensitive factors. The incidence rates of adverse events and associated costs curve fitting parameters of were found to be insensitive factors. Refer to Figure 3 for specific details.

**Figure 4:**
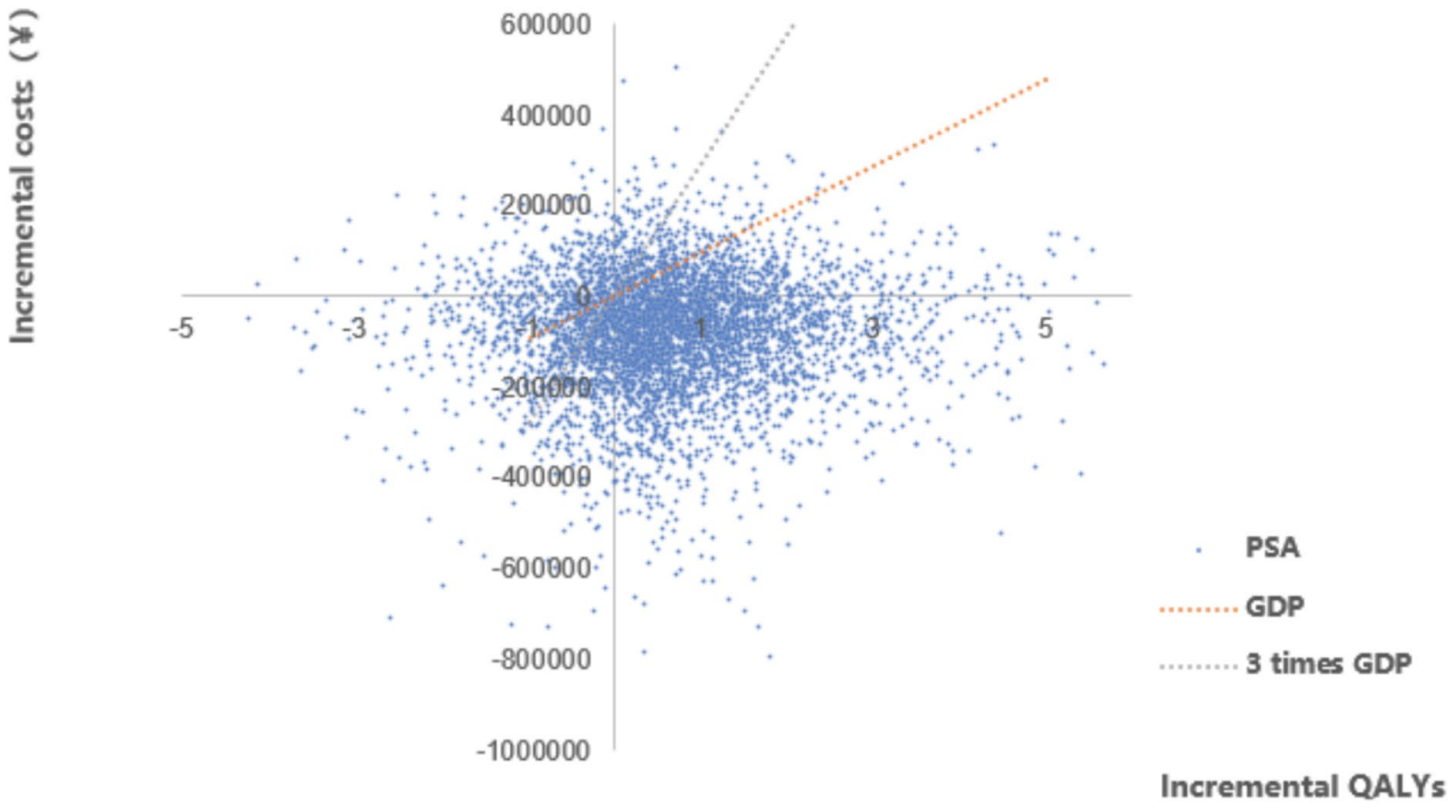
Scatter plot of the probabilistic sensitivity analysis results.

**Figure 5:**
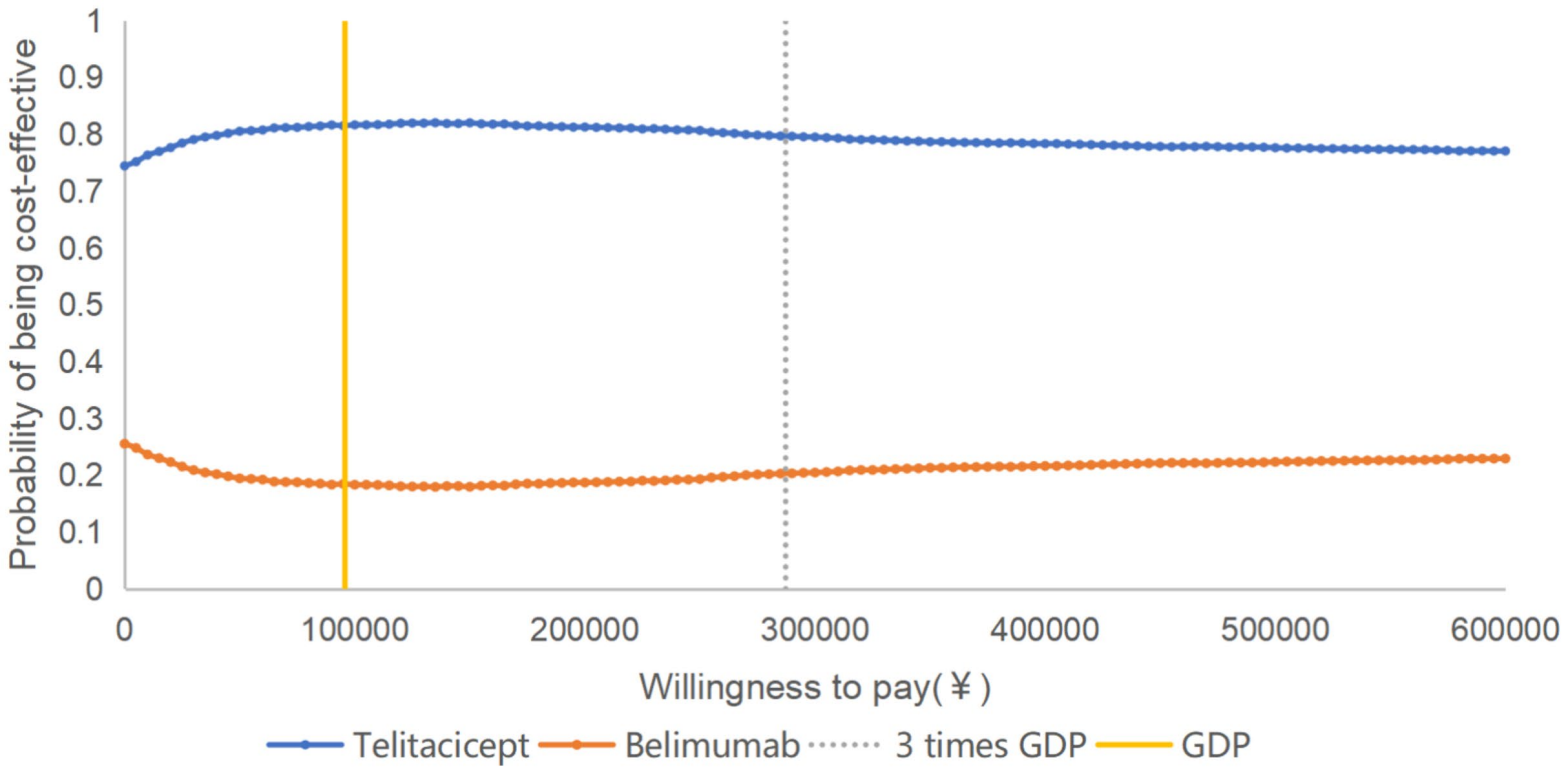
Cost-utility acceptability curve of telitacicept versus belimumab.

The PSA results showed that the majority of the data points were located in the fourth quadrant in the cost-utility plane (Figure 4), indicating lower costs and greater health benefits for the telitacicept group in most scenarios. According to the cost-utility acceptability curve (Figure 5), when the WTP per additional QALY is 1–3 times the per capita GDP of China, the probability of telitacicept being cost-effective remains approximately 80%.

## Discussion

In the treatment of SLE, innovative biologics enable glucocorticoid sparing with well-established efficacy. The Chinese Guidelines for the Diagnosis and Management of Systemic Lupus Erythematosus (2025 Edition) (7) explicitly recommend biologic therapy for patients with SLE who fail to achieve treatment targets or experience disease relapse after glucocorticoid and conventional immunosuppressant therapy. Telitacicept is a novel recombinant fusion protein of both the ligand-binding domain of the TACI receptor and the crystallizable fragment component of human IgG, which is a BLyS/APRIL dual inhibitor, demonstrates favorable therapeutic effects in autoimmune diseases (23).

Clinical studies (11–13) have demonstrated that biological agents are effective in treating SLE, significantly reducing disease activity. Model-based projections indicate long-term benefits, including reduced disease flares, organ damage, and mortality. Telitacicept shows slightly superior efficacy compared with belimumab in delaying disease progression and controlling disease activity. Consequently, the model simulations suggest that patients treated with telitacicept achieve greater health gains (approximately 0.5 QALYs) than those of receiving belimumab.

Regarding the telitacicept treatment duration, published clinical studies worldwide have used doses of either 160 mg or 80 mg per administration. The drug’s prescribing label explicitly states: “The recommended dose is 160 mg per administration. If dose reduction is required, the dose may be reduced to 80 mg per administration.” Clinical experts indicate that after approximately 6 months of treatment, when disease activity significantly decreases in active SLE patients, a dose reduction of half is commonly considered in clinical practice. Based on these comments, our study applied telitacicept’s dosage at the recommended 160 mg per administration for the first 6 months, followed by a reduced maintenance dose of 80 mg per administration thereafter. This dose reduction schedule resulted in lower total treatment costs for the telitacicept group compared to belimumab, which may better reflect real-world clinical practice.

The cost-utility analysis results show that telitacicept provides greater health gains compared to belimumab in SLE treatment by more effectively slowing disease progression. Despite higher initial costs from its longer full-dose regimen, Telitacicept results in lower long-term medication costs after dose reduction compared to belimumab, while also reducing organ damage management expenses, thereby exhibiting superior cost-effectiveness. Scenario analysis results indicate that the total healthcare costs between the two treatment groups become equivalent at approximately 3.08 years.

This study has several limitations: (1) as biological agents for SLE, including telitacicept and belimumab, remain relatively new in the Chinese clinical setting, real-world long-term experience with their use is still limited. Telitacicept has been newly included in the latest guideline (7) for the treatment of SLE, and the longest follow-up duration reported in existing studies about biological agents for SLE is limited to 52 weeks (11). Furthermore, their prescribing information does not specify a definitive treatment duration. Given the chronic and lifelong nature of SLE, which typically necessitates ongoing therapy to control disease progression, this study adopted a lifetime treatment horizon to calculate the cumulative cost difference between the two treatment strategies. This modeling choice, however, introduces extrapolation limitations, primarily because it does not account for potential long-term risks in real-world practice, such as diminished drug efficacy (or resistance), non-adherence, and delayed adverse events. Regarding these specific risks, clear evidence is currently lacking in the published literature. To address the uncertainty stemming from the assumption of sustained lifetime efficacy—which may not fully reflect real-world discontinuation patterns—scenario analyses for short term treatment duration was conducted. Additionally, extensive sensitivity analyses were performed to test the robustness of the model’s outcomes. (2) The efficacy inputs were sourced from real-world studies, in which the baseline characteristics and background therapies of the two groups were comparable. Therefore, other than the specific biological agent used, treatments such as glucocorticoid therapy, immunosuppressants, or treatment switching, were assumed to be identical between the groups and were thus excluded from the model calculation. (3) Due to data availability constraints and comparable medication scenarios between the two groups, cost differentials pertaining to healthcare utilization, such as basic treatment, medical examination and hospitalization expenditures were excluded from the analysis. (4) Multiple studies have reported health utility values for SLE patients from different perspectives (24–27). As the model was constructed based on the SLEDAI-2 K, only health utility values directly linked to SLEDAI-2 K scores were incorporated into the calculations. This approach, which did not comprehensively consider all utility values reported in the literature, may introduce a potential source of bias into the study findings. (5) Key efficacy parameters were derived from meta-analysis of three currently published real-world studies comparing telitacicept versus belimumab. The lack of head-to-head clinical trials requires further validation of the efficacy differences between these agents. Future large-sample, high-quality RCTs and related epidemiological studies are recommended to verify these model findings.

## Conclusion

Under current pricing, treatment settings and efficacy data, telitacicept demonstrates superior cost-effectiveness compared with belimumab for long-term SLE management by reducing medication costs while delivering additional health benefits.

## Data Availability

The original contributions presented in the study are included in the article/Supplementary material, further inquiries can be directed to the corresponding author.
