# Supplementary material for: Cost-utility analysis of telitacicept versus belimumab in the treatment of systemic lupus erythematosus in China
Source: Front Public Health. 2026 Jan 15;13:1712454. doi: 10.3389/fpubh.2025.1712454 (PMC12852384; doi:10.3389/fpubh.2025.1712454)
Supplement: Supplementary file 1 [file Table_1.docx]

Table 1 Calculation of monthly organ damage and death probabilities of different disease activities

| Disease activity | General organ damage | | Death | |
| --- | --- | --- | --- | --- |
|  | Rate | Coefficient | Rate | Coefficient |
| Baseline (7 years) | | | | |
| Not activity | 39.000% | 9.000% | 8.000% | 22.000% |
| Estimated (monthly) | | | | |
| Not activity | 0.587% | 0.112% | 0.099% | 0.295% |
| Mild | 0.619% |  | 0.100% |  |
| Moderate | 0.763% |  | 0.104% |  |
| Severe | 1.235% |  | 0.111% |  |

Note: Since SLEDAI was used in this study, the rules for classifying disease activities are different from SLEDAI-2K.

Table 2 Calculation of utility values of different disease activities

| SLEDAI-2K | UTILITY | coefficient | Disease activity | median |
| --- | --- | --- | --- | --- |
| Baseline value | | | | |
| 5.2 | 0.67 | -0.010 |  |  |
| Estimated value | | | | |
| 0 | 0.7168 |  | Mild | 0.6898 |
| 1 | 0.7078 |  | Mild |  |
| 2 | 0.6988 |  | Mild |  |
| 3 | 0.6898 |  | Mild |  |
| 4 | 0.6808 |  | Mild |  |
| 5 | 0.6718 |  | Mild |  |
| 6 | 0.6628 |  | Mild |  |
| 7 | 0.6538 |  | Moderate | 0.6313 |
| 8 | 0.6448 |  | Moderate |  |
| 9 | 0.6358 |  | Moderate |  |
| 10 | 0.6268 |  | Moderate |  |
| 11 | 0.6178 |  | Moderate |  |
| 12 | 0.6088 |  | Moderate |  |
| 13 | 0.5998 |  | Severe | 0.5683 |
| 14 | 0.5908 |  | Severe |  |
| 15 | 0.5818 |  | Severe |  |
| 16 | 0.5728 |  | Severe |  |
| 17 | 0.5638 |  | Severe |  |
| 18 | 0.5548 |  | Severe |  |
| 19 | 0.5458 |  | Severe |  |
| 20 | 0.5368 |  | Severe |  |

Note: Since the maximum SLEDAI-2K score recorded in the included clinical trials did not exceed 20, health utility values were calculated only for the score range of 0 to 20.

Table 3 Distribution settings in probabilistic sensitivity analysis

| Parameters | Distribution | α/Lower value | β/Upper value |
| --- | --- | --- | --- |
| **Curve fitting parameters** |  |  |  |
| Telitacicept group baseline (0-week) distribution - Mean | Normal | 9.3329 | 10.5760 |
| Telitacicept group baseline (0-week) distribution - SD | Normal | 0.3858 | 0.4372 |
| Telitacicept group endpoint (24-week) distribution - Mean | Normal | 2.8604 | 3.2415 |
| Telitacicept group endpoint (24-week) distribution - SD | Normal | 0.7309 | 0.8283 |
| Belimumab group baseline (0-week) distribution - Mean | Normal | 9.3329 | 10.5760 |
| Belimumab group baseline (0-week) distribution - SD | Normal | 0.3858 | 0.4372 |
| Belimumab group endpoint (24-week) distribution - Mean | Normal | 3.4335 | 3.8908 |
| Belimumab group endpoint (24-week) distribution - SD | Normal | 0.2301 | 0.2607 |
| **Damage events** |  |  |  |
| Organ damage incidence (Mild activity) | Beta | 95.44 | 15321.16 |
| Organ damage incidence (Moderate activity) | Beta | 95.30 | 12387.24 |
| Organ damage incidence (Severe activity) | Beta | 94.84 | 7585.35 |
| Mortality rate (Mild activity) | Beta | 95.94 | 95469.18 |
| Mortality rate (Moderate activity) | Beta | 95.94 | 92185.53 |
| Mortality rate (Severe activity) | Beta | 95.93 | 85997.09 |
| **Utilities** |  |  |  |
| Utility value (Mild activity) | Beta | 29.10 | 13.09 |
| Utility value (Moderate activity) | Beta | 34.78 | 20.31 |
| Utility value (Severe activity) | Beta | 40.89 | 31.06 |
| Organ damage disutility | Beta | 68.65 | 174.61 |
| **Costs** |  |  |  |
| Unit price of Telitacicept(￥/vial) | Gamma | 96.04 | 8.10 |
| Unit price of Belimumab(￥/vial) | Gamma | 96.04 | 7.29 |
| Adverse event rate (Telitacicept) | Beta | 64.33 | 132.57 |
| Adverse event rate (Belimumab) | Beta | 64.02 | 129.97 |
| Adverse event management cost (￥) | Gamma | 96.04 | 0.81 |
| Organ damage treatment cost (￥) | Gamma | 96.04 | 63.49 |
| **Discount rates** |  |  |  |
| Discount rate (Costs) | Uniform | 0 | 8% |
| Discount rate (Effects) | Uniform | 0 | 8% |
